# The PRC2-associated factor C17orf96 is a novel CpG island regulator in mouse ES cells

**DOI:** 10.1038/celldisc.2015.8

**Published:** 2015-04-28

**Authors:** Robert Liefke, Yang Shi

**Affiliations:** 1 Division of Newborn Medicine and Program in Epigenetics, Department of Medicine, Boston Children’s Hospital, Boston, MA, USA; 2 Department of Cell Biology, Harvard Medical School, Boston, MA, USA

**Keywords:** Chromatin, ES cells, Histone Modifications, PRC2, CpG Islands

## Abstract

CpG islands (CGIs) are key DNA regulatory elements in the vertebrate genome and are often found at gene promoters. In mammalian embryonic stem (ES) cells, CGIs are decorated by either the active or repressive histone marks, H3K4me3 and H3K27me3, respectively, or by both modifications (‘bivalent domains’), but their precise regulation is incompletely understood. Remarkably, we find that the polycomb repressive complex 2 (PRC2)-associated protein C17orf96 (a.k.a. esPRC2p48 and E130012A19Rik) is present at most CGIs in mouse ES cells. At PRC2-rich CGIs, loss of C17orf96 results in an increased chromatin binding of Suz12 and elevated H3K27me3 levels concomitant with gene repression. In contrast, at PRC2-poor CGIs, located at actively transcribed genes, C17orf96 colocalizes with RNA polymerase II and its depletion leads to a focusing of H3K4me3 in the core of CGIs. Our findings thus identify C17orf96 as a novel context-dependent CGI regulator.

## Introduction

A large proportion of mammalian promoters are characterized by a cluster of CpG dinucleotides, commonly known as CpG islands (CGIs). Work in the past decades has revealed that CGIs have fundamental roles in gene regulation, and their misregulation is associated with cancer [1]. Most CpG dinucleotides in the genome are cytosine-5 methylated, but at CGIs, CpGs sites typically remain unmethylated, but acquire methylation during disease processes such as cancer, resulting in gene silencing [1–3]. Proteins that specifically recognize either methylated or unmethylated CpG have been identified to participate in the regulation of CGI activities. For example, Cfp1 and Kdm2b recognize unmethylated CGIs and are involved in the establishment of the active histone H3 lysine 4 trimethylation mark (H3K4me3) [4] or H2A ubiquitination [5], respectively, while MBP proteins bind to methylated CpGs [6, 7]. In mammalian embryonic stem (ES) cells, the polycomb repressive complex 2 (PRC2) is typically found at CGIs, but the underlying link between CGIs and PRC2 remains elusive. It has been proposed that potentially an unknown factor or H2A ubiquitination has a role in the recruitment of PRC2 to CGIs [3, 5, 8, 9]. Further, it has been demonstrated that PRC2 is recruited to artificially introduced CGIs in the genome, but not when those CGIs are associated with transcription [10, 11], suggesting that active transcription prevents PRC2 recruitment. Consistently, inhibition of transcription is sufficient to rapidly increase the PRC2 occupancy at CGIs and for *de novo* recruitment of PRC2 to CGI sites [12], but the underlying mechanism is unclear.

Recent biochemical purifications of PRC2 followed by mass spectrometry analysis identified novel interaction partners of PRC2 (our unpublished data, and Zhang *et al. *[13], Smits *et al. *[14] and Hunkapiller *et al. *[15]). One of these factors, C17orf96 (a.k.a. esPRC2p48 and E130012A19Rik), has been found to be a main interacting partner of the mammalian PRC2 (our unpublished data and Smits *et al.* [14]). C17orf96 has evolved in mammals and is highly expressed in ES cells, brain and during embryogenesis (Supplementary Figure S1) [16–18], and has been described to be a downstream target of the pluripotency factor Klf5 [19]. C17orf96 has been suggested to have a critical role during somatic cell reprogramming and neuronal differentiation processes [13, 16], but its mechanism of action remains elusive. In contrast to other PRC2 interaction partners, C17orf96 is intrinsically unstructured and it does not possess any domain or structure of known function [20].

Here, we demonstrate that C17orf96 is localized genome wide at CGIs in mouse ES (mES) cells, irrespective of their transcriptional status. At PRC2-rich CGIs, C17orf96 negatively regulates PRC2 and H3K27me3 levels. At PRC2-poor CGIs, C17orf96 depletion leads to a redistribution of H3K4me3. These findings identify C17orf96 as a novel CGI-associated protein, which modulates histone modifications at CGIs through regulating histone modifying enzyme complexes such as PRC2.

## Results

### PRC2-rich and PRC2-poor CGIs are equally bound by C17orf96 in mES cells

**Figure 1:**
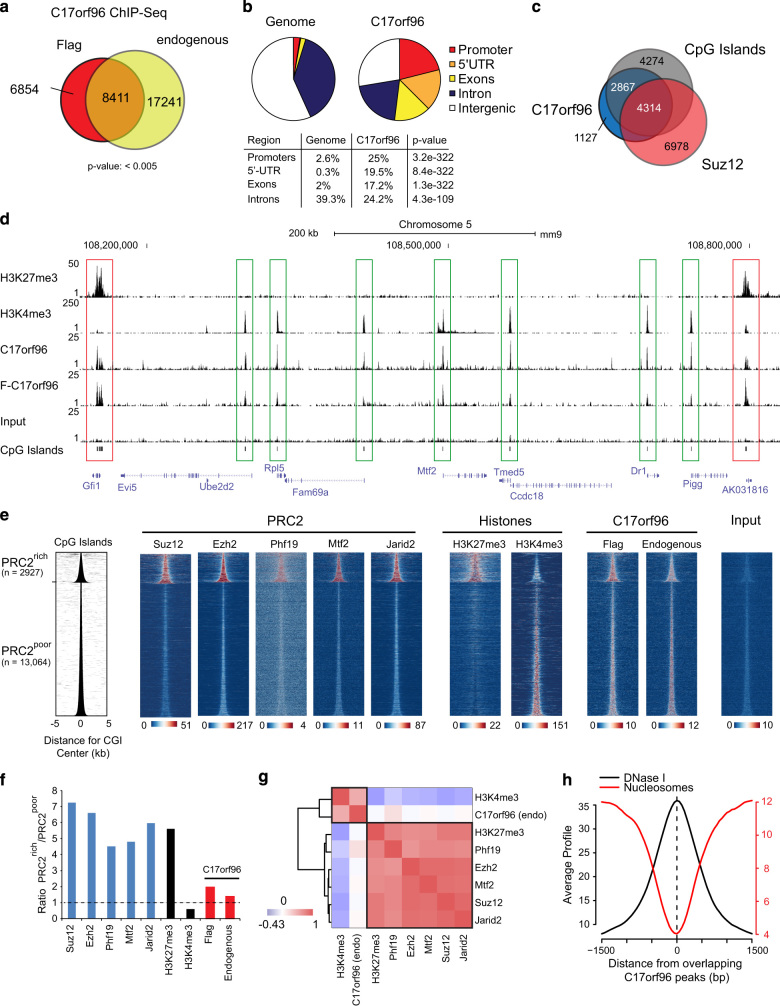
C17orf96 is present at PRC2-rich and PRC2-poor CGIs. (**a**) Overlap of significant peaks (MACS, *P*<10^−5^) of ChIP-seq experiments performed in mES cells against Flag-mC17orf96 (using M2 beads) or the endogenous protein using a home-made antibody (Supplementary Figure S2). Overlapping peaks are considered has high-confident C17orf96-bound location. (**b**) C17orf96 is enriched at promoter regions, but depleted from intergenic regions. (**c**) C17orf96 peaks overlaps strongly with CGIs, but less with the PRC2 core component Suz12. (**d**) Example UCSC genome browser view, demonstrating that C17orf96-bound CGIs can be occupied by the repressive H3K27me3 [40] and active H3K4me3 histone marks. (F-C17orf96=Flag-C17orf96). (**e**) CGIs were categorized into PRC2-rich and PRC2-poor CGIs, based on the Suz12 data set [41]. Heatmaps of known PRC2 members [33, 42–44] at CGIs show a predominant presence at PRC2-rich CGIs, which correlates with presence of H3K27me3 [40] and reduced level of H3K4me3. C17orf96 is almost equally distributed between both groups. (**f**) Based on the average profiles that ratio of the factors between PRC2-rich /PRC2-poor CGIs has been calculated. The Flag-tagged C17orf96 protein is more dominantly bound at PRC2-rich CGIs than the endogenous protein. (**g**) Calculation of correlation coefficients between endogenous C17orf96, PRC2 members, H3K27me3 and H3K4me3 at CGIs demonstrates that C17orf96 correlates strongest with H3K4me3. (**h**) C17orf96 occupied locations are depleted for nucleosomes [45] and possess enhanced DNase I hypersensitivity [46].

Previously, while purifying PHF19, we identified C17orf96 as a major interacting component, which co-migrates with the PRC2 complex in glycerol gradient fractionation experiments. Importantly, our biochemical purification of C17orf96 also led to the co-purification of the entire PRC2 complex (data not shown), implicating C17orf96 as a component of the PRC2 complex, which is consistent with the published findings reported recently [13–15, 21]. To investigate the molecular function of C17orf96 in mES cells, we determined the genomic locations of both the endogenous (Supplementary Figure S2) as well as an N-terminal Flag-tagged, overexpressed C17orf96 by chromatin immunoprecipitation followed by sequencing (ChIP-seq). We found a significant overlap between the two data sets (Supplementary Figure S3A), that is, both antibodies identified a total of 8411 overlapping peaks (Figure 1a), which we consider as high confidence sites. These sites are strongly enriched at promoter regions, but depleted from intergenic regions (Figure 1b). Interestingly, C17orf96 peaks only partially overlap with regions bound by the PRC2 core member Suz12. Instead, they strongly overlap with CGIs, independent of whether they are decorated by repressive or active histone marks. (Figures 1c and d). Notably, one of the strongest peaks of the endogenous C17orf96 can be found at a large CGI at its own gene (*E130012A19Rik*; Supplementary Figure S3B).

To determine the relationship between C17orf96, PRC2 and CGIs in more depth, we bioinformatically defined two categories of CGIs: CGIs that are rich in PRC2 (*n*=2917) and CGIs that are weakly bound by PRC2 (*n*=13 064). Remarkably, while all other PRC2 components are only strongly enriched at PRC2-rich CGIs, C17orf96, although an interacting protein of PRC2 [13, 14, 21], is almost equally distributed between these two groups (Figures 1e and f). In line with this observation, at CGIs, C17orf96 correlates better with the active histone mark H3K4me3 than with PRC2 and H3K27me3 (Figure 1g). Further analysis showed that C17orf96 binds to locations that are depleted for nucleosomes and are sensitive to DNase I digestion, suggesting that C17orf96 preferentially binds to nucleosome-free regions at CGIs (Figure 1h). To confirm the ChIP-seq results, we performed ChIP experiments followed by quantitative PCR at several CGI-containing genes. Consistently, we found C17orf96, as well as Suz12, H3K27me3 and H3K4me3, more strongly enriched at locations with CGIs than at nearby locations without CGI (Supplementary Figure S4). In sum, our ChIP-seq results in mES cells revealed an unanticipated localization of C17orf96 not only at CGIs occupied by PRC2 but also at CGIs that are associated with actively transcribed genes. This finding supports a potentially global role of C17orf96 at CGIs, as well as a function of C17orf96 that is independent of PRC2.

### C17orf96 depletion elevates H3K27me3 at PRC2-rich CGIs

**Figure 2:**
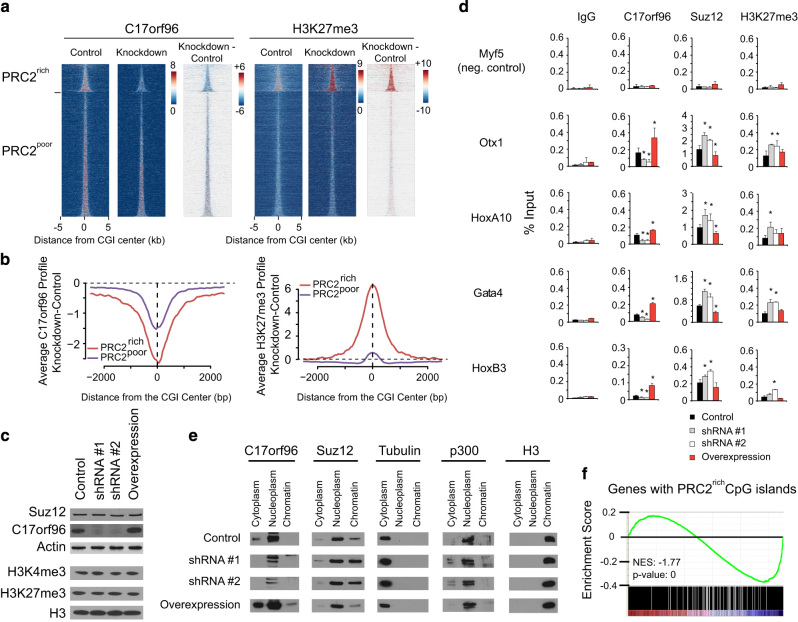
C17orf96 depletion affects Suz12 chromatin binding and H3K27me3 deposition. (**a**) Heatmap at CGIs (as in Figure 1e) of the ChIP-seq of C17orf96 and H3K27me3 in mES cells infected with scrambled shRNA (‘control’) or a specific shRNA for C17orf96 (shRNA #2; ‘knockdown’). The last heatmap for each antibody shows the subtraction of the knockdown versus control cells. (**b**) Differential profiles of knockdown versus control. Knockdown of C17orf96 reduces C17orf96 signal at PRC2-poor and PRC2-rich CGIs, but increases the H3K27me3 specifically at PRC2-rich CGIs. (**c**) Western blot analysis of four created cell lines, infected with a construct expressing control shRNA, two specific shRNA for mouse C17orf96 and mouse C17orf96 (untagged; ‘overexpression’). No global change of Suz12, H3K4me3 and H3K27me3 could be detected. See also Supplementary Figure S2. (**d**) ChIP experiments on genes with PRC2-rich CGIs, using the four cell lines described. Knockdown of C17orf96 enhances Suz12 and H3K27me3 levels. Overexpression reduces Suz12 levels, but does not appear to affect H3K27me3. Values represent the average and s.d. of two independent experiments. *P<0.05. (**e**) Cellular fractionation of the four cell lines. The level of chromatin-bound Suz12 negatively correlates with the level of C17orf96. Notably, C17orf96 is mostly in the nucleoplasm and cytoplasm, but hardly in the chromatin fraction. See also Supplementary Figure S5D. (**f**) Gene set enrichment analysis of genes possessing a PRC2-rich CGI using microarray data from De Cegli *et al. *[16]. Knockdown of C17orf96 leads to a significant reduced expression of those genes.

To further elucidate the role of C17orf96 at CGIs, we created stable mES cells lines expressing either a C17orf96 short hairpin RNA (shRNA; shRNA #2, ‘knockdown’) or a scrambled shRNA (‘control’), respectively. As expected, upon knockdown of C17orf96 expression, ChIP-seq revealed ~50% average reduction in occupancy at CGIs (Figures 2a and b, and Supplementary Figure S5A). However, a small portion of C17orf96 peaks at highly expressed genes with low nucleosome and high RNA polymerase II occupancy remained relatively constant (Supplementary Figures S5B and C), possibly because a fraction of C17orf96 remained bound to chromatin even in the presence of C17orf96 shRNA (Supplementary Figure S5D). Importantly, upon C17orf96 knockdown, H3K27me3 was increased at CGIs that are occupied by PRC2, but showed no overt changes at PRC2-poor CGIs (Figures 2a and b). These results suggest that reduction of C17orf96 enhances the action of PRC2 at PRC2-rich CGIs, which leads to an increased deposition of H3K27me3. To confirm, we performed additional ChIP experiments in stable mES cells where C17orf96 was knocked down by two independent shRNAs (shRNA #1 and 2) or cells overexpressing an untagged mouse C17orf96 (‘overexpression’; Figure 2c and Supplementary Figure S2). Consistent with our ChIP-seq results, knockdown of C17orf96 leads to an increase of H3K27me3 as well as an increase of Suz12, a core component of PRC2. In contrast, overexpression of C17orf96 results in a reduction of Suz12, but interestingly no significant change of H3K27me3 (Figure 2d). Given that the overall protein level of Suz12 was unaltered in response to C17orf96 knockdown (Figure 2c), these results suggest that C17orf96 might interfere with the binding of PRC2 to chromatin. Consistently, biochemical fractionation experiments showed that the level of chromatin-bound Suz12 exhibits a negative correlation with the level of C17orf96 (Figure 2e). Published microarray data [16] demonstrated that knockdown of C17orf96 has only mild effects on gene expression, which is consistent with the observation that removal of PRC2 has only subtle impacts on the transcriptional program in ES cells [12]. However, upon performing gene set enrichment analysis [22] we found that genes that possess a PRC2-rich CGI are significantly more often downregulated in the C17orf96 knockdown cells (*n*=1368, normalized enrichment score: −1.77, *P*-value=0; Figure 2f), compared with genes with a PRC2-poor CGI (*n*=7553, normalized enrichment score: −0.85, *P*-value=0.99) or genes with no CGI (*n*=5235, normalized enrichment score: 1.51, *P*-value=0).

**Figure 3:**
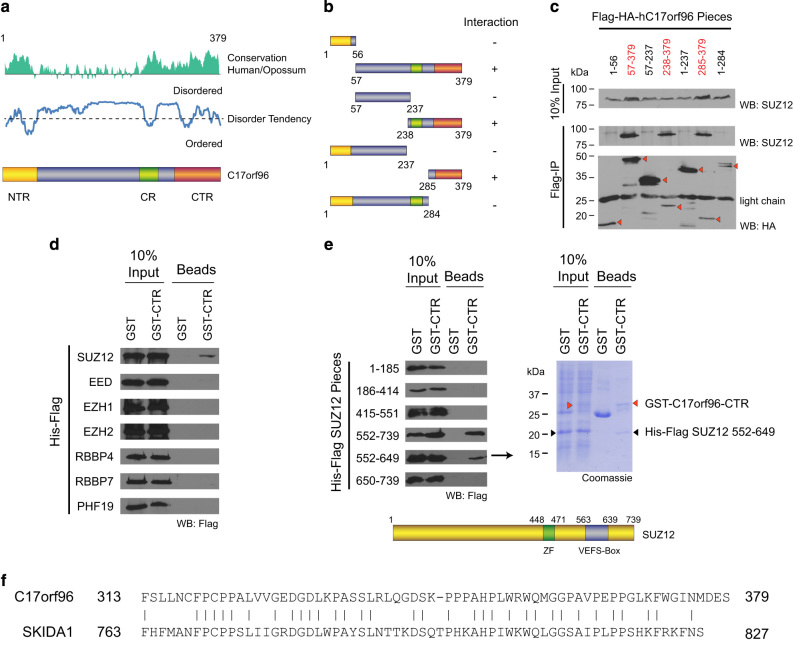
C17orf96 directly interacts with SUZ12. (**a**) C17orf96 is an intrinsically unstructured protein but it possesses three regions (N-terminal region (NTR), central region (CR) and C-terminal region (CTR)) with enhanced homology and reduced disorder tendency, suggesting functional relevance. The homology plot was obtained using Vector NTI by comparing C17orf96 from human and opossum. The disorder blot was made using IUPreD [47] with human C17orf96. (**b**, **c**) Semi-endogenous co-immunoprecipitation in HeLa-S cells expressing different parts of human C17orf96. The CTR is necessary and sufficient to co-immunoprecipitate the endogenous SUZ12 protein. (**d**, **e**) Using a bacterial co-expression approach followed by batch purification, the CTR (aminoacids 285–379) was found to directly interact with the VEFS-box of SUZ12. The Coomassie shows the interaction of GST–CTR, and not GST only, with the VEFS-box of SUZ12. (**f**) The CTR is conserved among C17orf96 and SKIDA1 (C10orf140; 52% identity).

To gain insight into the potential mechanism by which C17orf96 interferes with PRC2 function, we mapped the interaction of C17orf96 with PRC2. Via semi-endogenous co-immunoprecipitation, we identified the C-terminal region of C17orf96 as being necessary and sufficient for the interaction with PRC2 *in vivo* (Figures 3a–c), and found that this region associates with the VEFS-box of the PRC2 core member SUZ12 *in vitro* (Figures 3d and e), suggesting that C17orf96 may directly affect PRC2 chromatin binding by altering the functionality of Suz12 [23–25]. Notably, the C-terminal region has evolved in early vertebrates and is also present in the paralog SKIDA1 (C10orf140; Figure 3f), another novel PRC2-interacting protein [14], supporting that proteins possessing this sequence are PRC2 regulators in the entire vertebrate branch.

Taken together, these results suggest that C17orf96 inhibits PRC2 chromatin binding at PRC2-rich CGIs as evidenced by the observation that C17orf96 knockdown results in an elevated Suz12 occupancy, increased H3K27me3 levels and significantly, albeit moderately, reduced transcription of PRC2 target genes.

### C17orf96 depletion redistributes the H3K4me3 signal at PRC2-poor CGIs

**Figure 4:**
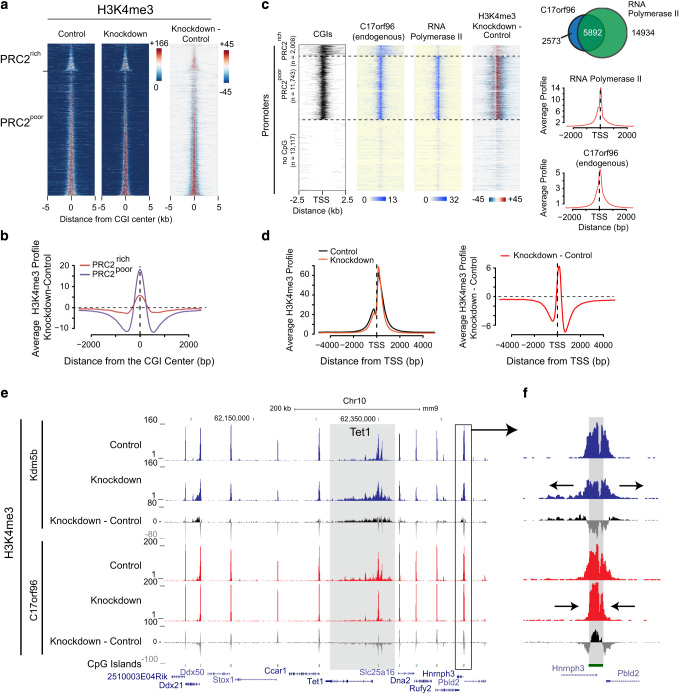
C17orf96 and Kdm5b knockdown affect H3K4me3 distribution oppositely. (**a**) Heatmaps of H3K4me3 at PRC2-rich and PRC2-poor CGIs as described in Figure 2a. Upon knockdown of C17orf96, H3K4me3 is increased in the core of CGIs but is reduced outside of CGIs. (**b**) Differential profiles of H3K4me3 knockdown versus control at PRC2-rich and PRC2-poor CGIs. The focusing is stronger at the PRC2-poor CGIs. (**c**) Heatmaps of C17orf96, RNA polymerase II and H3K4me3 change at gene promoters possessing a PRC2-rich, PRC2-poor or no CGI. At genes with PRC2-poor CGIs, C17orf96 localizes downstream of the transcription start site (TSS) similar to RNA polymerase II (8WG16) [48], which correlates with the strength of H3K4me3 redistribution. The Venn diagram shows the overlap of C17orf96 peaks (overlap from Flag and endogenous ChIP-seq peaks) and RNA polymerase II peaks. The promoter profiles of endogenous C17orf96 and RNA polymerase II show similarity. (**d**) Profiles of H3K4me3 in C17orf96 control and knockdown cells, showing the redistribution of H3K4me3. The profile of the difference indicates redistribution predominantly downstream of the TSS. (**e**) Genome browser view showing the comparison of H3K4me3 changes upon knockdown of Kdm5b [26] (blue) or C17orf96 (red). The effects are highly opposite at most CGIs. Strong effects are seen at broad H3K4me3 domains, such as at the *Tet1* gene locus (gray box), which are weakly occupied by C17orf96 (Supplementary Figure S6). (**f**) One example CGI, showing the opposite effects of Kdm5b and C17orf96 knockdown.

Next, we investigated the impact of C17orf96 knockdown on the active histone mark H3K4me3 (Figure 4). Surprisingly, our data revealed a major redistribution of the H3K4me3 mark upon C17orf96 knockdown, that is, an increase of H3K4me3 in the core of CGIs but a reduction outside of CGIs (Figures 4a and b). This change of H3K4me3 is mainly found at genes with PRC2-poor CGIs and is most apparent downstream of the transcription start site, where RNA polymerase II and C17orf96 show colocalization, suggesting a potential link to the transcription machinery (Figures 4a–d). Remarkably, an opposite phenomenon has been described for the knockdown of the histone H3K4me3 demethylase Kdm5b [26], a homolog of the trithorax group protein Lid (little imaginal disc) [27]. A comparison of the H3K4me3 changes after C17orf96 and Kdm5b knockdown, respectively, shows that C17orf96 and Kdm5b influences H3K4me3 in mES cells in an opposite manner (Figures 4e and f). This suggests that Kdm5b and C17orf96 may interfere with the same pathway that regulates H3K4me3 installation and/or spreading. Furthermore, the effect of knockdown of either Kdm5b or C17orf96 is most evident at the recently described, broad H3K4me3 domains [28], such as the *Tet1* gene locus (Figure 4e, gray box). These domains are occupied by C17orf96 (Supplementary Figure S6) and have been proposed to have a critical role to maintain transcriptional consistency of genes that define the cellular identity [28], suggesting that C17orf96 may be relevant to establishing the cellular identity during embryogenesis. Interestingly, the redistribution of H3K4me3 does not appear to have a major impact on gene expression of the associated genes (see above), which is in agreement with the previous observations that changes in H3K4me3 have low impact on transcription [29]. Taken together, these data further support that C17orf96 has a PRC2-independent role at CGIs, which affects the distribution of H3K4me3, directly or indirectly.

## Discussion

In the mammalian genome, ~50% of all promoters possess a CGI. Work in the past has lead to the discovery of several CGI-binding proteins, including Cfp1, Kdm2a/b, Tet1/3, Kmt2a/b and the MBD proteins, which are critical for the function of CGIs and for gene regulation. All those proteins are characterized by a CXXC zinc finger or an MBD domain that specifically recognizes either unmethylated or methylated CpGs, and most of them have a preference for CGIs that are either decorated with repressive or active marks [6, 7]. Importantly, this study discovers that C17orf96 associates with CGIs without a strong bias, suggesting that it is a general regulator of CGIs in mES cells (Figure 1). This idea is supported by published ChIP-seq data of N- and C-terminal tagged C17orf96 in human 293T cells [21], showing that also in differentiated cells many C17orf96 peaks overlap with CGIs (Supplementary Figure S7). In mES cells, C17orf96 differentially regulates histone modifications at CGIs that are linked to active or repressed transcription. Our findings thus identified a novel CGI regulator, which may function to fine-tune the chromatin state at CGI sites in mammalian ES cells.

**Figure 5:**
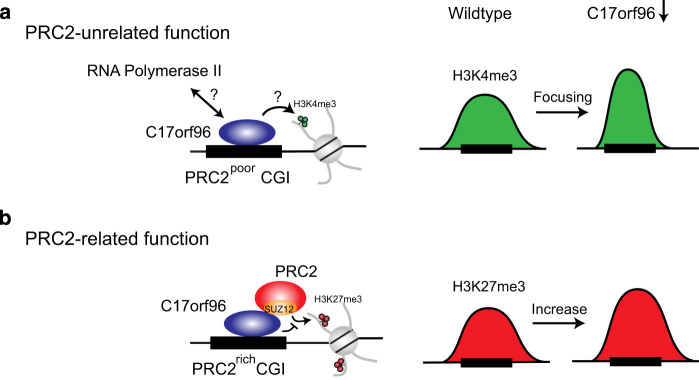
Model of function of C17orf96 at CGIs. (**a**) At CGIs that are not or hardly occupied by PRC2 (PRC2-poor), C17orf96 depletion leads to a focusing of H3K4me3 at the core of CGIs. At those CGIs, C17orf96 also colocalizes with RNA polymerase II suggesting a potential link to the transcription machinery. (**b**) At CGIs that are occupied by PRC2 (PRC2-rich), C17orf96 negatively affects the binding of PRC2 to chromatin. Consequently, upon knockdown of C17orf96, PRC2 recruitment and H3K27me3 deposition are enhanced.

C17orf96 does not contain a classical CpG-binding domain, suggesting that other yet-to-be-identified mechanisms are responsible for its recruitment to CGIs. Of note, in a nucleosomal pulldown experiment C17orf96 has been found to be associated with unmethylated but not with DNA methylated nucleosomes, suggesting that DNA methylation may be of relevance for C17orf96 recruitment to CGIs [30]. As C17orf96 is an intrinsically unstructured protein (Figure 3a) [20], insights into how C17orf96 is recruited to CGIs will possibly reveal novel mechanisms important for understanding CGI functions. Although C17orf96 was initially identified as a PRC2-interacting protein, our work revealed both a PRC2-associated and PRC2-independent role of C17orf96 (Figure 5). At CGIs where there is virtually no PRC2, loss of C17or96 causes redistribution of H3K4me3 (Figure 4). Given that C17orf96 colocalizes with RNA polymerase II (Figures 4c and d, and Supplementary Figures S5B and C), the function of C17orf96 at those CGIs could conceivably be linked to transcriptional activity (Figure 5a). Notably, the PRC2 subunits Ezh1, Ezh2 and Suz12 have also been demonstrated to colocalize with RNA polymerase II and H3K4me3 [31–33], supporting a complex interplay between PRC2 components and the transcription machinery at active genes.

At the PRC2-rich CGIs, C17orf96 modulates the amplitude of gene transcription by interfering with H3K27 trimethylation as depletion of C17orf96 not only causes an increase in H3K27me3 level but also an elevated recruitment of PRC2 (Figures 2 and 5b). Our biochemical results suggest that C17orf96 directly interacts with Suz12, a core subunit of the PRC2 complex (Figures 3d and e), which is important for chromatin binding of PRC2 [23–25]. Further investigation of the interplay between C17orf96 and PRC2 will provide additional insights into the role of C17orf96 regarding PRC2.

In ES cells, many CGIs that are occupied by PRC2 and decorated by H3K27me3 also possess low level of H3K4me3 and form so-called bivalent domains, proposed to represent a poised transcriptional state [34, 35]. During differentiation, this poised state is resolved and the corresponding genes either become transcriptionally active or repressed [35]. Given that our study identified a role for C17orf96 in influencing the active H3K4me3 and the repressive H3K27me3 mark, it will be of interest to investigate in the future whether or not C17orf96 has a role to resolve bivalent domains upon differentiation and the underlying the molecular mechanism.

In summary, we have identified C17orf96 as a putative, novel CGI regulatory protein in mES cells whose roles appear to modulate histone modification patterns at CGIs through influencing the functions of chromatin modifying machineries such as PRC2.

## Materials and Methods

### Plasmids

The ORF for mouse C17orf96 (E130012A19Rik) was cloned via PCR from mES cell cDNA. The ORF from human C17orf96 were synthesized from GeneScript. The ORF for human PHF19, RBBP4, RBBP7, EZH1 and EZH2 were cloned via PCR using cDNA from HeLa-S cells. The SUZ12 construct was a kind gift from Danny Reinbergs laboratory. Lentiviral shRNA constructs for mouse C17orf96 were created by cloning hairpins (shRNA #1: 5ʹ-GGAGCATCGATTCTGAAATTT-3ʹ and shRNA #2: 5ʹ-ATGATGGAAGATGGAATAAAT-3ʹ) into the pLKO.1 vector. SHC202 (Sigma-Aldrich, St Louis, MO, USA) was used as shRNA control. Quantitative PCR primers for mouse C17orf96 are presented in Supplementary Table S3.

### Antibodies

The antibody against mouse C17orf96 was generated by immunizing rabbits (Covance, Princeton, NJ, USA) with a peptide corresponding to the N terminus of mouse C17orf96 (LKPRRGTPEFSPLC). Sera were obtained and the antibodies were positively selected using a purified GST-PLKPRRGTPEFSPLCL fusion protein and negatively selected using bacterial crude cell extract, each coupled to CNBr-activated Sepharose beads. Other used antibodies were Suz12 (Cell Signaling, no. D39F6 (ChIP); Santa Cruz, Dallas, TX, USA, sc-46264/sc-271325 (Western)), p300 (Santa Cruz, sc-585), Tubulin (Sigma-Aldrich, T9026), actin (Abcam, Cambridge, MA, USA, ab3280), histone H3 (Abcam, ab1791), H3K27me3 (EMD Millipore, Billerica, MA, USA, no. 07-449), H3K4me3 (EMD Millipore, no. 04-745), Flag M2 (Sigma-Aldrich, F1804), Flag M2 beads (Sigma-Aldrich, A2220) and HA.11 (MMS-101P, Covance).

### ChIP and ChIP-seq

ChIP experiments were performed via crosslinking ChIP as described [36]. Flag ChIP was performed using Flag M2 beads from Sigma. ChIP-seq libraries were constructed of 10 ng of ChIP DNA following Illumina’s protocol and sequenced using an Illumina Genome Analyzer (Ilumina, San Diego, CA, USA). ChIP DNA were quantified using gene-specific primers (Supplementary Table S2) with the LightCycler 480 II (Roche, Basel, Switzerland).

### Bioinformatics analysis

Own and public ChIP-seq data (Supplementary Table S1) were mapped to mouse genome mm9 or human hg19 using bowtie version 1.0 [37], allowing one mismatch (*n*=1) and maximal three possible alignments (*m*=3). All subsequent analyses of ChIP-seq data were performed using the Cistrome pipeline [36, 38]. PRC2-rich and PRC2-poor CGIs were defined using the k-means clustering function of the heatmap tool in Cistrome. For heatmaps, wiggles obtained from Model-based Analysis for ChIP-Seq (MACS) (standard settings) were used. The subtraction wiggles (Figures 2 and 4) were obtained using a custom workflow in Cistrome. For normalization, the data set with more ChIP-seq tags were downsized to the smaller data set before MACS. For promoter definition, RefSeq genes were downloaded from the UCSC Table Browser. After removal of duplicates with identical transcription start site, 26 840 promoters, including genes with alternative transcription start sites, were used for analysis. CGIs were downloaded from the UCSC Table Browser. For the correlation analysis, ChIP-seq tags were counted at each individual CGI using a custom R script for Bioconductor [39]. Peaks for C17orf96, RNA polymerase II and Suz12 were called via MACS with a cutoff *P*-value of 1e−05. Microarrays [16] were normalized via Robust Multi-array Average (RMA) using Bioconductor. Gene set enrichment analysis [22] were performed with standard settings.

### Cell culture

E14 mES cells (ES-E14TG2a) were cultured in dulbecco’s modified eagle medium (DMEM), 15% fetal calf serum (FCS), 1×l-glutamine (Gibco, Life Technologies, Grand Island, NY, USA), 1×non-essential amino acids (Gibco), 1×sodium pyruvate, 1×penicillin/streptavidin (Gibco), 0.15% β-mercaptoethanol and leukemia inhibitory factor (LIF) (EMD Millipore, no. ESG1107) on gelatin-coated plates. Stable cell lines were created via infection with lentiviral vectors harboring the appropriate construct and selected via puromycine (1 μg ml^−1^). HeLa-S and 293T cells were cultured with DMEM, 10% FCS and 1×penicillin/streptavidin (Gibco).

### Cellular fractionation

Cellular fractionation was perfomed using ‘Subcellular Protein Fractionation Kit for Cultured Cells’ (Pierce, no. 78840) according to manufacturer’s instructions, followed by western blotting.

### Co-immunoprecipitation

Stable HeLa-S cells expressing Flag-HA-tagged pieces of human C17orf96 were obtained via lentiviral infection and puromycine selection. Whole-cell extracts were made using CHAPS buffer (Tris 50 mm, pH 7.8, 350 mm NaCl, 1 mm dithiothreitol (DTT) and 10 mm CHAPS). For immunoprecipitation, samples were incubated with anti-Flag M2 beads (Sigma-Aldrich) for 3 h at 4 °C. After washing the beads with CHAPS buffer, the precipitated proteins were visualized by western blotting.

### Co-expression-coupled GST pulldown

BL21 Gold cells were transfected with two constructs, a bait construct expressing a glutathion-S-transferase (GST) fusion protein of the C-terminal region (aminoacids 285–379) of C17orf96 or GST only and a prey construct expressing a His-Flag-tagged protein from a PRC2 member or from a SUZ12 fragment. The cells were selected with Kanamycin and Ampicillin. The proteins were co-expressed at 16 °C and 100 mm Isopropyl-β-D-thiogalactopyranosid (IPTG) and after cell lysis the GST fusion protein was coupled to glutathione beads for 2 h. After three times washing using CHAPS buffer (see above), the binding of the His-Flag-tagged protein was visualized by western blotting using a Flag antibody.

### Immunofluorescence

mES cells were plated on gelatin-treated coverslips for 1 day. Cells were fixed with 3.2% paraformaldehyde for 10 min, washed with wash buffer (1× PBS containing 0.5% NP-40), then incubated with blocking buffer (wash buffer with 10% fetal bovine serum), and stained with rabbit mC17orf96 antibody for 2 h diluted in blocking buffer. Secondary antibody (goat anti-rabbit Alexa Fluor 594) was obtained from EMD Millipore.

### Statistical analyses

The significance of the data was either calculated by Cistrome, the gene set enrichment analysis software or via unpaired Student’s *t*-tests.

### Data deposition

ChIP-seq data are available under the GEO accession number GSE63491.
