# Supplementary material for: The PRC2-associated factor C17orf96 is a novel CpG island regulator in mouse ES cells
Source: Cell Discov. 2015 Apr 28;1:15008–. doi: 10.1038/celldisc.2015.8 (PMC4860827; doi:10.1038/celldisc.2015.8)
Supplement: Supplementary Information [file celldisc20158-s1.doc]

**Supplementary Table 1:** Published Datasets used for bioinformatics analysis.

| **Protein** | **GEO ID** | **Reference** |
| --- | --- | --- |
| Mtf2 | GSM415050 | 1 |
| Jarid2 | GSM491760 | 2 |
| Suz12 | GSM1019771 | 3 |
| Phf19 | GSM1020086 | 4 |
| Ezh2 | GSM1199182/GSM1199183 | 5 |
| H3K27me3 | GSM307619 | 6 |
| RNA Polymerase II | GSM749807 | 7 |
| DNase I | GSM1300602 | 8 |
| Nucleosomes | GSM1004653 | 9 |
| H3K4me3 Control | GSM1282127/GSM1282137 | 10 |
| H3K4me3 KDM5B shRNA | GSM1282129/GSM1282139 | 10 |
| C17orf96 (N), 293T | GSM1294939 | 11 |
| C17orf96 (C), 293T | GSM1294937 | 11 |
| C10orf12, 293T | GSM1294933 | 11 |
| EZH2, 293T | GSM1294929 | 11 |
| Input, 293T | GSM1294938 | 11 |

**1 Walker E, Chang WY, Hunkapiller J *et al.* Polycomb-like 2 associates with PRC2 and regulates transcriptional networks during mouse embryonic stem cell self-renewal and differentiation. *Cell Stem Cell* 2010; 6:153-166.**

**2 Li G, Margueron R, Ku M, Chambon P, Bernstein BE, Reinberg D. Jarid2 and PRC2, partners in regulating gene expression. *Genes Dev* 2010; 24:368-380.**

**3 Ballare C, Lange M, Lapinaite A *et al.* Phf19 links methylated Lys36 of histone H3 to regulation of Polycomb activity. *Nat Struct Mol Biol* 2012; 19:1257-1265.**

**4 Brien GL, Gambero G, O'Connell DJ *et al.* Polycomb PHF19 binds H3K36me3 and recruits PRC2 and demethylase NO66 to embryonic stem cell genes during differentiation. *Nat Struct Mol Biol* 2012; 19:1273-1281.**

**5 Kaneko S, Son J, Shen SS, Reinberg D, Bonasio R. PRC2 binds active promoters and contacts nascent RNAs in embryonic stem cells. *Nat Struct Mol Biol* 2013; 20:1258-1264.**

**6 Mikkelsen TS, Ku M, Jaffe DB *et al.* Genome-wide maps of chromatin state in pluripotent and lineage-committed cells. *Nature* 2007; 448:553-560.**

**7 Lin C, Garrett AS, De Kumar B *et al.* Dynamic transcriptional events in embryonic stem cells mediated by the super elongation complex (SEC). *Genes Dev* 2011; 25:1486-1498.**

**8 Sherwood RI, Hashimoto T, O'Donnell CW *et al.* Discovery of directional and nondirectional pioneer transcription factors by modeling DNase profile magnitude and shape. *Nat Biotechnol* 2014; 32:171-178.**

**9 Teif VB, Vainshtein Y, Caudron-Herger M *et al.* Genome-wide nucleosome positioning during embryonic stem cell development. *Nat Struct Mol Biol* 2012; 19:1185-1192.**

**10 Kidder BL, Hu G, Zhao K. KDM5B focuses H3K4 methylation near promoters and enhancers during embryonic stem cell self-renewal and differentiation. *Genome Biol* 2014; 15:R32.**

**11 Alekseyenko AA, Gorchakov AA, Kharchenko PV, Kuroda MI. Reciprocal interactions of human C10orf12 and C17orf96 with PRC2 revealed by BioTAP-XL cross-linking and affinity purification. *Proc Natl Acad Sci U S A* 2014; 111:2488-2493.**

**Supplementary Table 2:** ChIP qRT-PCR primers

|  |  |
| --- | --- |
| Msx1 CpG f | ACAGAAAGAAATAGCACAGACCATAAGA |
| Msx1 CpG r | TTCTACCAAGTTCCAGAGGGACTTT |
| Msx1 noCpG f | TTTGTCGGGCACAAATACAA |
| Msx1 noCpG r | CGCTGGAATACAGTTGAGCA |
| Otx1 CpG f | AGTAGGCGTGCTCAGAGAGG |
| Otx1 CpG r | GGCCGGTCAAGAAGAAGTC |
| Otx1 noCpG f | CCCTGACCAACTCCAGTCTC |
| Otx1 noCpG r | GCCAGAAGTTCAGCCATCTC |
| HoxA10 CpG f | CTTTTGCGCAGAACATCAAA |
| HoxA10 CpG r | GTAGCCGGGTACTGGCACT |
| HoxA10 noCpG f | CTCCGGCCTTTCATTCTCTC |
| HoxA10 noCpG r | CCGGGTCTTTCCCATTTAGT |
| Sox2 CpG f | CCATCCACCCTTATGTATCCAAG |
| Sox2 CpG r | CGAAGGAAGTGGGTAAACAGCAC |
| Sox2 noCpG f | TGCAGGTTGATATCGTTGGT |
| Sox2 noCpG r | GGCAGCCTGATTCCAATAAC |
| Myf5 f | GGAGATCCGTGCGTTAAGAATCC |
| Myf5 r | CGGTAGCAAGACATTAAAGTTCCGTA |
| Gata4 f | AAGAGCGCTTGCGTCTCTA |
| Gata4 r | TTGCTAGCCTCAGATCTACGG |
| HoxB3 f | CCGTCGCATGAAGTACAAGA |
| HoxB3 r | CCTTAAGAGGGGGCTGGTAG |

**Supplementary Table 3:** qRT-PCR primers

| mGAPDH f | CGTCCCGTAGACAAAATGGTGA |
| --- | --- |
| mGAPDH r | GTAGACTCCACGACATACTCAG |
| mC17orf96 f | CCGGCTGATGCTCTTTCTAC |
| mC17orf96 r | AAACAACCGGTTTGTCCATC |


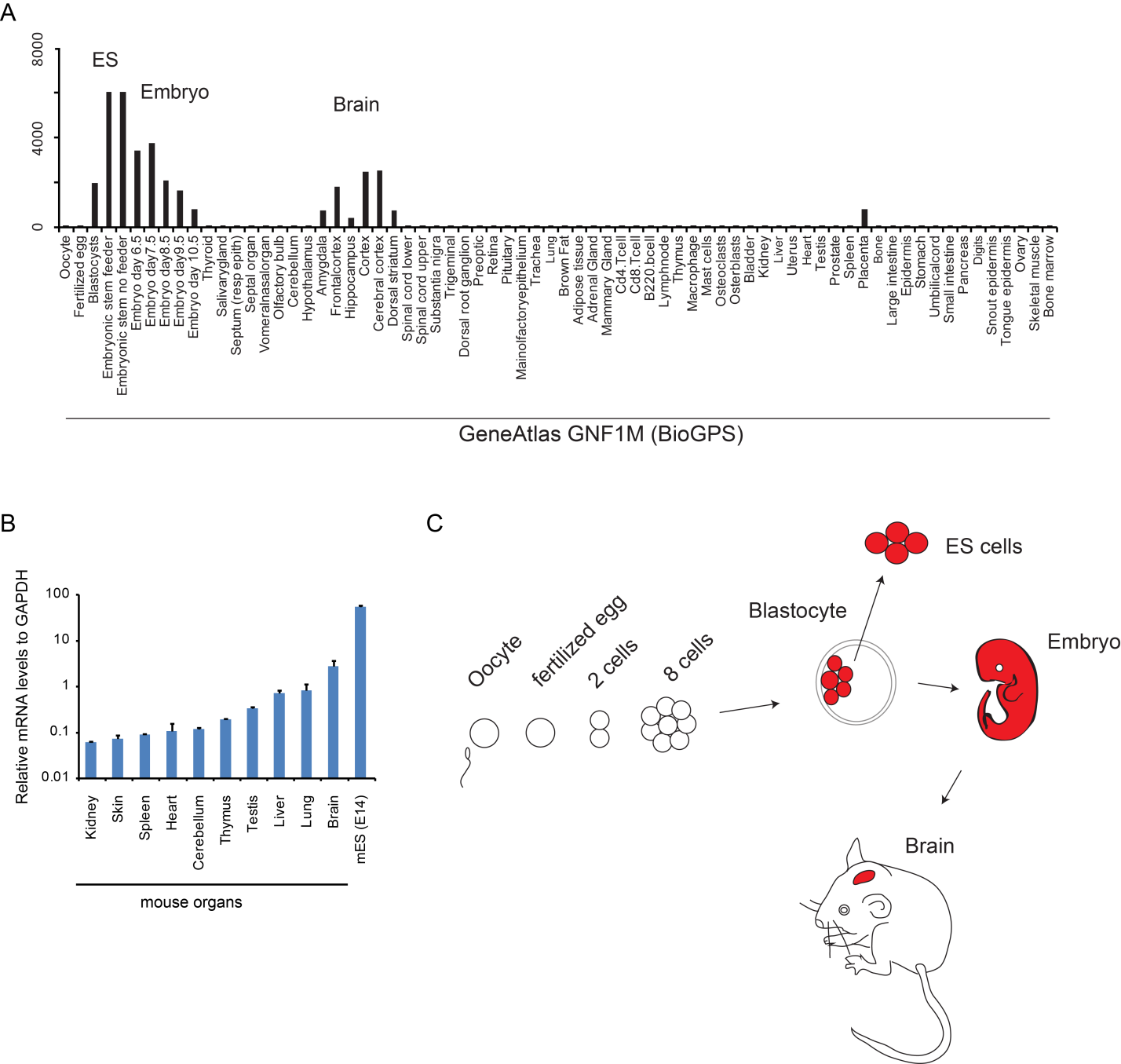


**Supplementary Figure 1:** A) BioGPS data show that C17orf96 is predominantly expressed in ES cells, embryo and in the adult brain. B) Quantitative real-time PCR on mouse organ cDNA and mES cells support strongest expression of C17orf96 in Brain and ES cells. C) Graphical overview of C17orf96 expression (red).


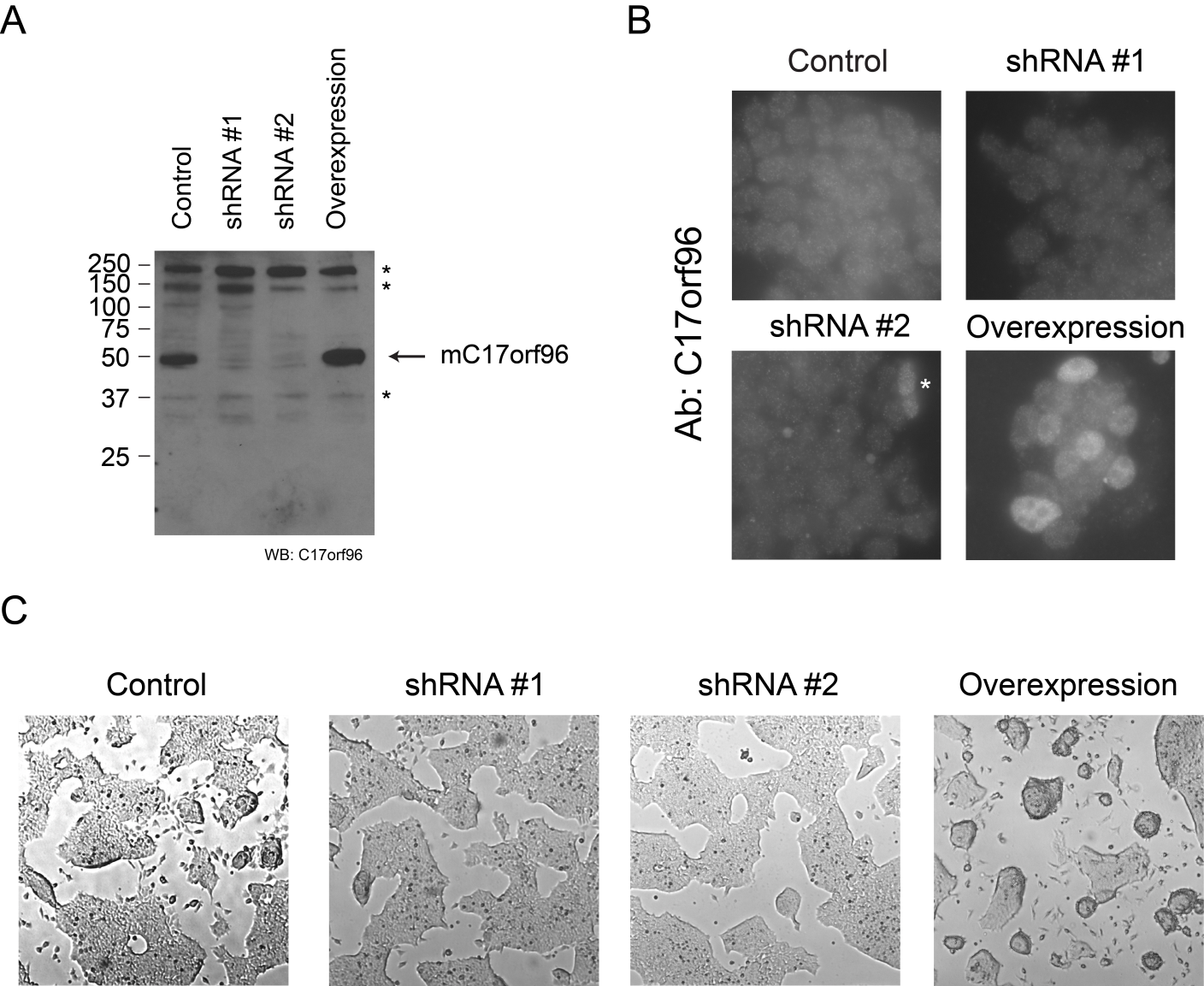


**Supplementary Figure 2:** A) Full C17orf96 Western blot of mES cells with C17orf96 knockdown or overexpression (Fig. 2C). Asterisks indicate unspecific bands. B) Immunofluorescence of the cells using the home-made antibody. The asterisk indicates two (rare) cells that have escaped the knockdown. C) Bright field microscopy of the four cell lines. Spontaneous differentiation of the mES cells positively correlates with the level of C17orf96.


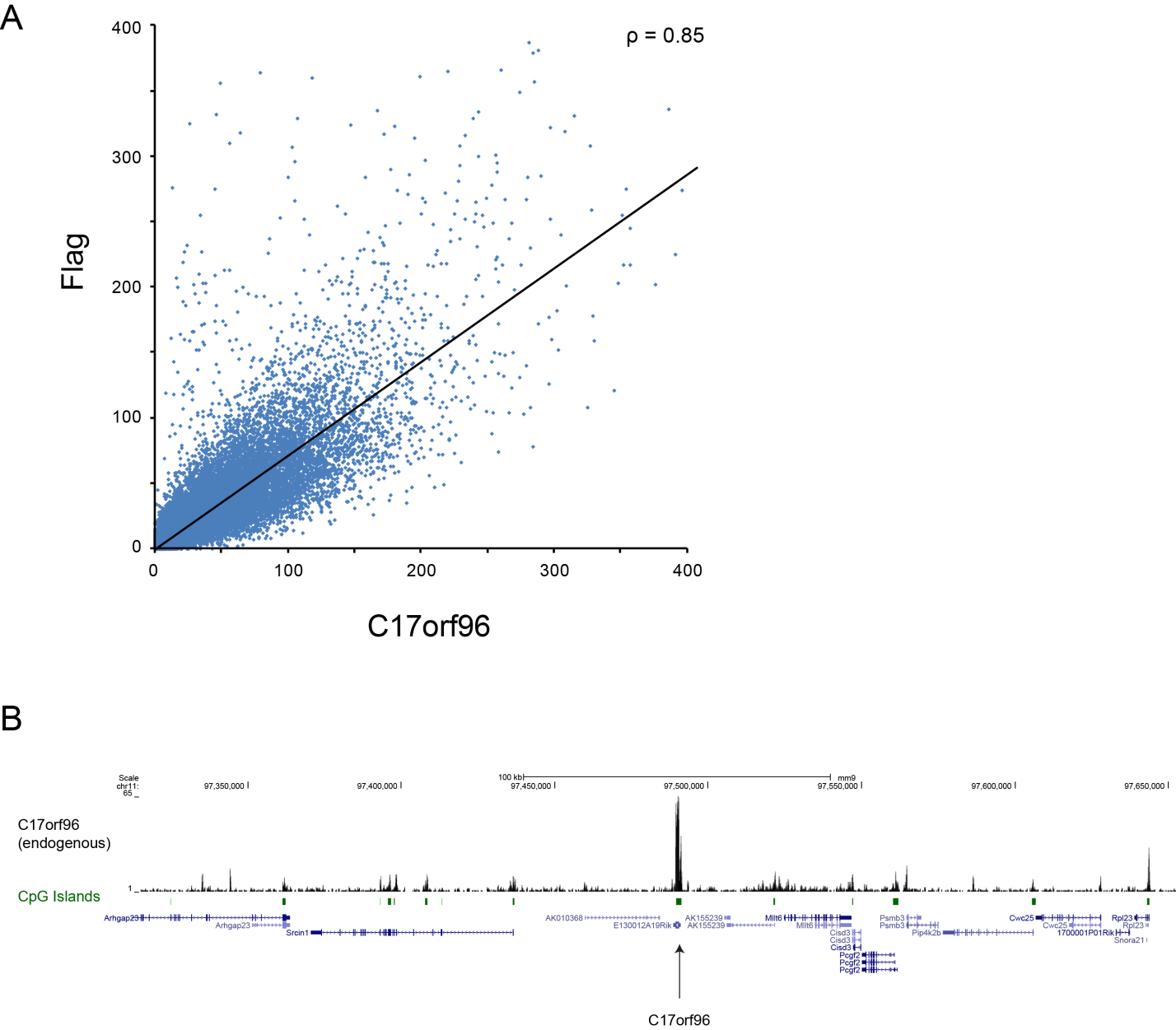


**Supplementary Figure 3:** A) Correlation of ChIP-Seq signals (tags per individual CGI) of Flag and endogenous ChIP. The datasets correlate with a spearman’s correlation coefficient of 0.85. B) Genome Browser view of the endogenous C17orf96 ChIP-Seq around the C17orf96 gene (E130012A19Rik).


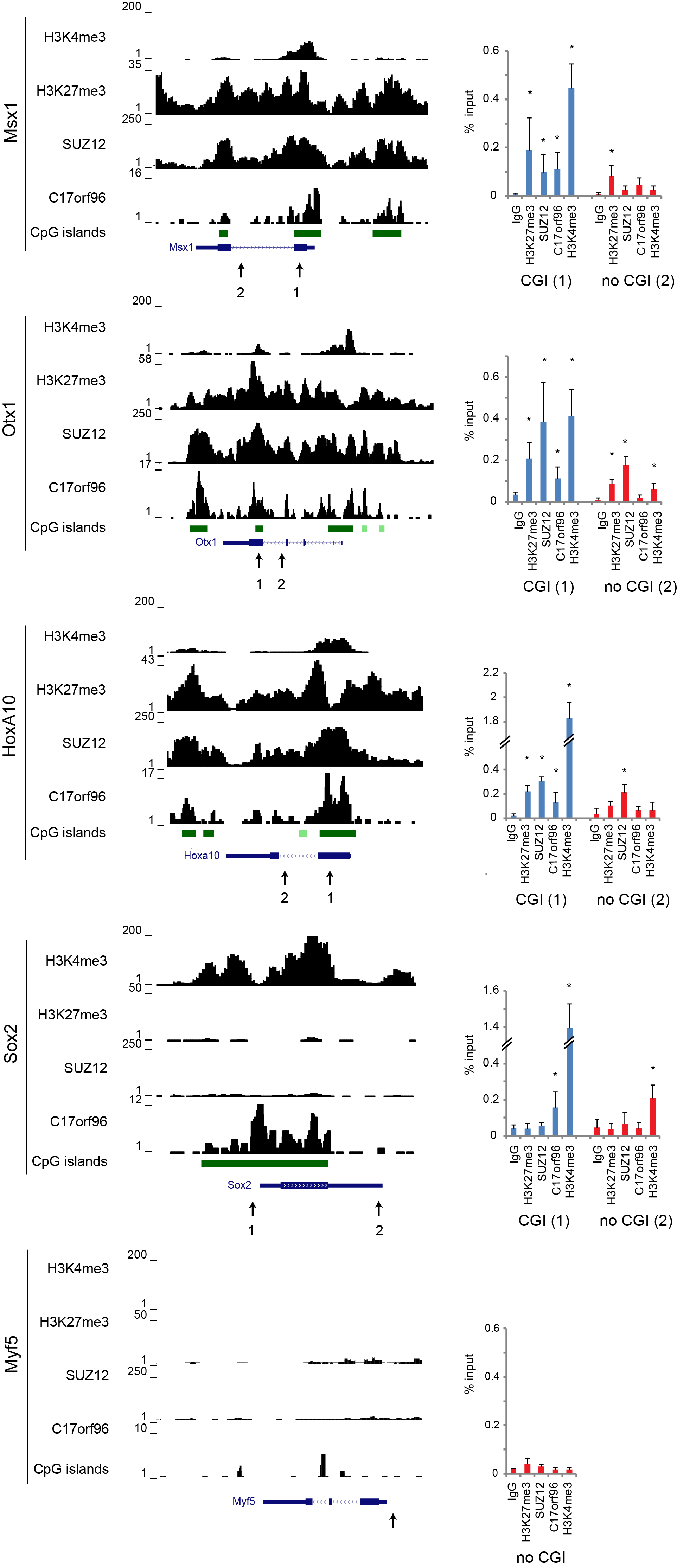


**Supplementary Figure 4:**

Validating ChIP experiments of the localization of C17orf96 at CpG islands. Values represent the average and s.d. of two independent experiments. * = p < 0.05

**
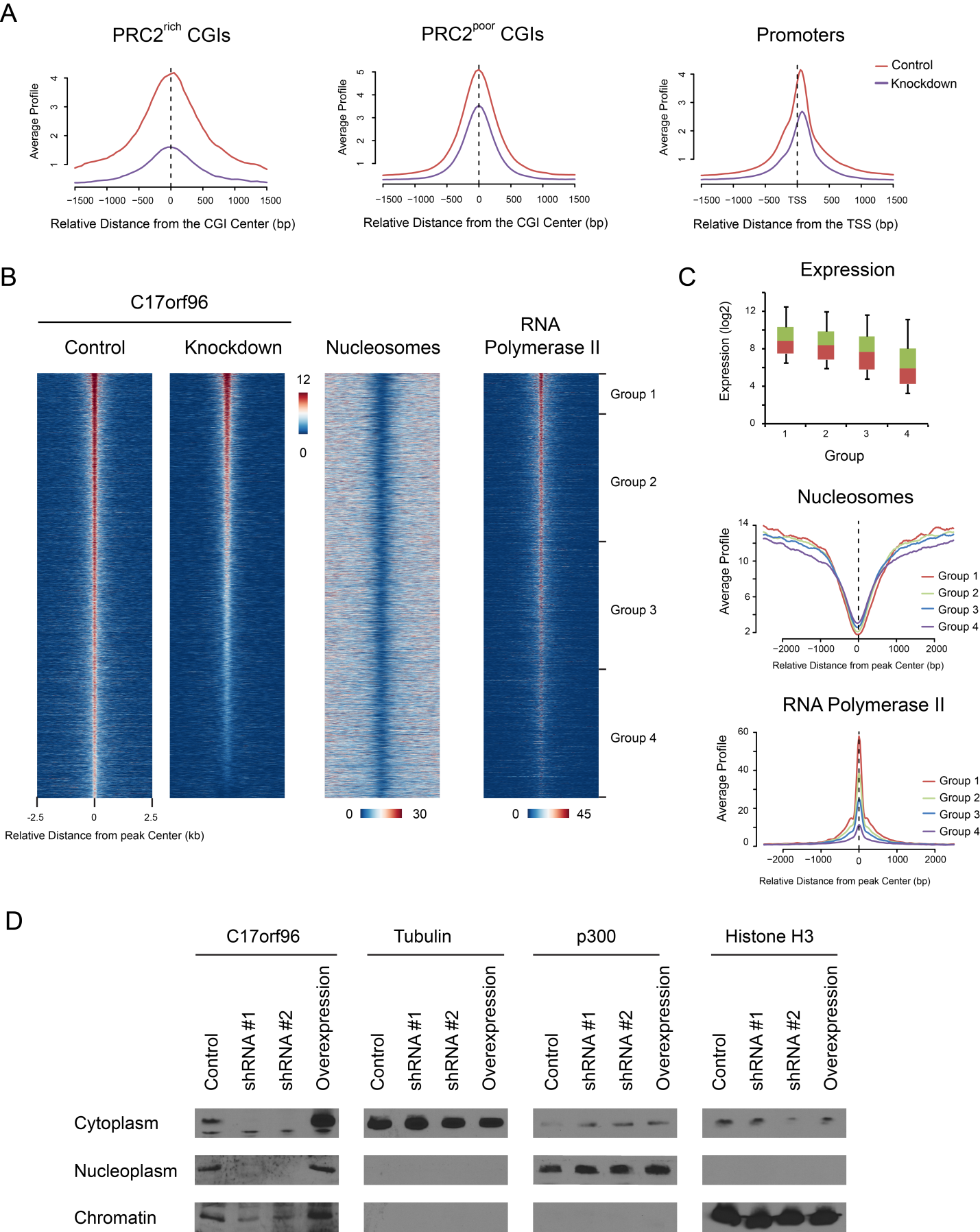
**

**Supplementary Figure 5:** A) ChIP-Seq profiles of C17orf96 at PRC-rich and PRC2-poor CGIs as well as gene promoters in control and knockdown cells. B and C) Upon knockdown of C17orf96, some peaks at places with low nucleosome and high RNA polymerase II occupancy, concomitant with high gene expression, remained relatively constant (group 1), suggesting that these locations are the preferred targets of C17orf96 under knockdown conditions. All heatmaps were sorted after the C17orf96 signal in the knockdown sample. C) Expression levels and profiles of nucleosomes and RNA Polymerase II in the four indicated groups from B). D) Analysis of C17orf96 presence in cytoplasm, nucleoplasm and chromatin fraction after knockdown and overexpression in mES cells. The protein level of C17orf96 in the chromatin fraction is least affected upon knockdown. Overexpression leads to an accumulation of C17orf96 in the cytoplasm.


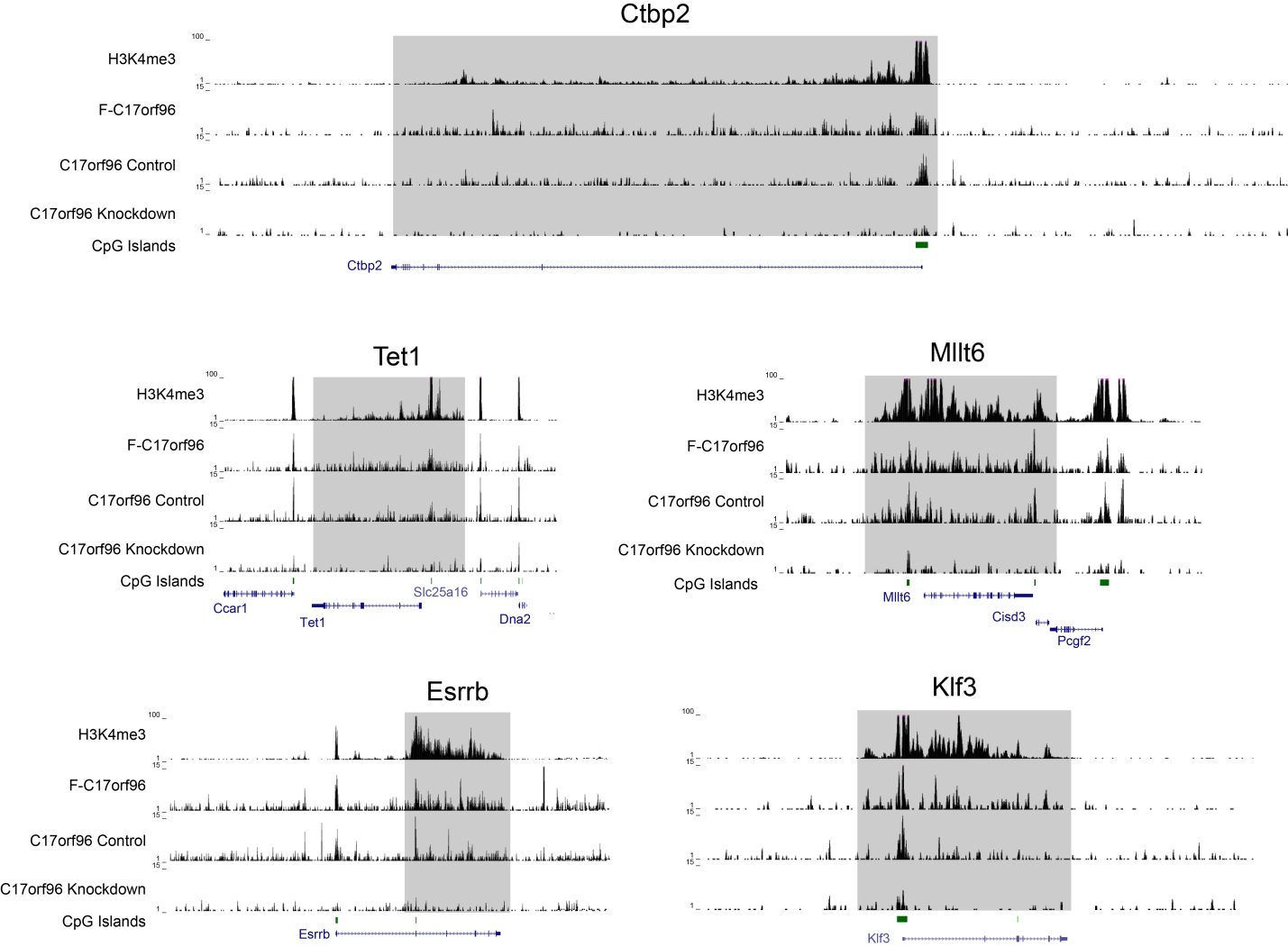


**Supplementary Figure 6:** Examples for C17orf96 occupancy at broad H3K4me3 domains (grey box).


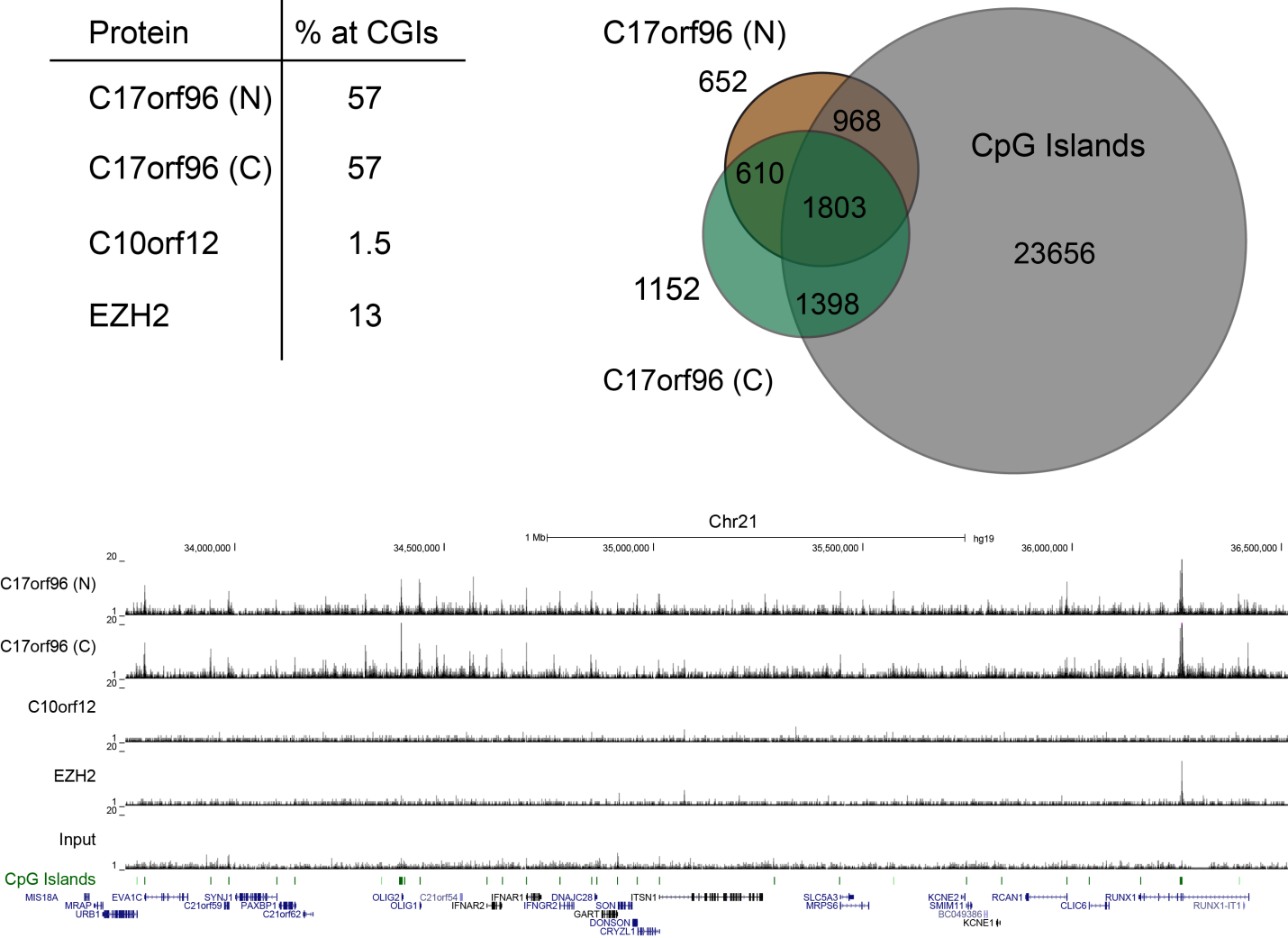


**Supplementary Figure 7:** ChIP-Seq data of C17orf96 in human 293T cells show overlap with CGIs.
